# A business process re-engineering approach to transform business process simulation to BPMN model

**DOI:** 10.1371/journal.pone.0277217

**Published:** 2023-03-15

**Authors:** Reema Choudhary, Nauman Riaz

**Affiliations:** Department of Computer Science, University of Gujrat, Gujrat, Punjab, Pakistan; University of Pisa, ITALY

## Abstract

Software reverse engineering and reengineering are becoming common in the field of games and website development. Simulation and modeling play an important role in understanding the flow of the overall system. Business process modeling notation (BPMN) is used to show the overall architecture of the business process. Simulated business process re-engineering is essential for implementing change or creating new processes. The simulation model explains whether a change will be successful or not prior to adopting any new business processes or other changes. Some available tools help convert the BPMN to a simulating BPMN model but converting the discrete event simulation model build in commercial off the shelf simulation packages like Simul8 to the BPMN to help generate business process simulation to BPMN is also a key challenge. This framework is introduced to convert the simulation model to BPMN using the reverse engineering concept to understand how the converting tools convert the BPMN model to the simulation model. After understanding this process, the concept of reengineering will be used to build a BPMN from the simulation model. The framework is divided into three main parts model translation, model mapping, and model formation. For model building, two simulation tools Simul8 and BPSimulator are used. It is then tested on two case studies bank and product manufacturing. The output shows the BPMN model is generated from the simulation model within less time on a single click saving time and resources for developing BPMN model first and then making simulation model for testing purpose.

## Introduction

Software reverse engineering is used to understand how the website or software code is developed by analyzing its process. Reverse engineering helps software engineers to understand the underlying process of the developed system. From the product to back, the process is what the reverse engineers can give [1]. Once the developers understand the process, they can reengineer it to build a similar product or code. This concept of reverse engineering and reengineering is the main motivation for this framework development.

Business process management (BPM) is the practice of analyzing, classifying, modifying, and monitoring business processes to ensure that, they run smoothly and may be improved over time [2]. System modeling is done by different graphical methods like a flowchart, Petri nets, data flow diagram, role activity diagram, business use-cases, BPMN. To represent the business process, BPMN has worldwide acceptance, which is increasing continuously now a day. BPM tools are intensively used in organizations [3]. Today’s accepted business process modelling standard is the BPMN. It is regarded as being unclear by all types of users, including business analysts who draught the initial versions of business processes, technical developers who write the software applications that will carry out those processes, and business professionals who will oversee and manage them. BPMN can be used to create both executable and natural business process models, such as those required for business process simulation [4].

A simulation is an imitation of the operation of a real-world process or system over time. Simulation is the dynamic view of the complex systems that need to be analyzed before the actual implementation of the system. Any environment can be visualized through a simulation model before taking the risk of an actual implementation. Developing a simulation helps in understanding the system. Moreover, for the systems before implementation or change, the simulation helps the business stakeholders to check whether the change or new procedure in the business is helpful or not. Simulation saves both cost and time by identifying any gap in the process [5].

Reengineering business processes becoming increasingly popular these days for developing new processes or changing existing ones. After analyzing the business model, changes are made to the business process as necessary. Results of the alteration prior to implementation are shown through BPR and simulation. The model is modified if the suggested adjustment is advantageous; otherwise, it is run again with modified parameters until the analysts discover results that are helpful [6]. Reverse engineering, often known as back engineering, is the practise of disassem bling software, equipment, aircraft, architectural structures, and other products in order to learn how they were made. Disassembling smaller pieces of bigger things is commonly an element of reverse engineering. By applying the reverse engineering method to discover how an item was made, you can recreate it [7].

Business analysts create BPMN models as part of the reengineering of business processes, while simulation practitioners use these models to create simulation models. To create a simulation model, various technologies that simulate BPMN are available. These technologies are not interoperable, and modelling complicated processes is impossible. We created a framework that can be used to create a BPMN model based on a simulation model using either a built-in simulation-modelling tool like Simul8 or a BPMN modelling tool like BPSimulator to help business analysts save time and money. The business process of simulation will be mapped to BP notations by first creating the XML from the simulation model and then extracting the data. It creates a BPMN model using these BP notations. In this study, business process simulations are automated into business process modelling notations (BPMN) using XML as a medium. It pulls the simulation model’s notations from the XML file and converts them to BPMN.

The remaining sections of the paper are organized as follows: background studies, knowledge of business process management, system modelling tools, business process re-engineering and simulation, BPMN and simulation, and discussion of reverse engineering. Following a discussion of the research methodology, the experiment is carried out on two case studies of banks and product manufacturing in the following section. In Simul8 and BPSimulator, the simulation model is created for both case studies. The output is then analyzed in BPMN format. The paper’s conclusion and suggested directions for future research are then covered.

## Background

### Business Process Management (BPM)

By identifying opportunities for new business ventures, outsourcing, increasing business efficiency, and locating areas within the company where technology can be used to support business processes, the fields of Business Process Modeling (BPM) and Reengineering aim to better understand a business’s key mechanisms in order to improve, and in some cases radically change, the business performance. Different authors define business process management as steps to discover, analyze, model and then optimize. As with earlier software modelling techniques, BPM methods have evolved over the past 20 years from a range of fields, but they share with those techniques a lack of standard procedures and notations, with each BPM method utilizing its own notation.

According to [8] the author defines a business process as, a business process is a group of actions that takes one or more types of input and produces an output that is valuable to the client. A business process has a goal and is impacted by events that take place within or outside of it. Another definition in [9] a business process focuses more on how work is carried out than it does on the goods or services that come as a result of the process. A process, according to [10], is "just a structured set of operations intended to create a specified result for a specific customer or market." In contrast to a product focus on what is done, it suggests a significant emphasis on how work is done inside a company. Thus, a process is an intentional arrangement of work activities through time and space with a start and end and clearly defined inputs and outputs: a framework for action.

### Modeling tools and methods

Different methods represent the system process in order to understand the business process. Developers use these methods or graphical notation to understand the flow of the system or the business process to show the system’s workflow. All the methods used for representing the process have their own described methods and notations. In this section, first a short description about the methods are discussed and then in Table 1 we compare the available modeling methods in terms of their usability, flexibility, and understandability for the user. Lastly, it is checked whether it can be converted to a simulation model or not. Following is a short description of the methods, which is then compared in the Table 1.

**Table 1:** BPMN notations with its description.

Notation	Description
Start Event	Each process must begin with a starting event. All start events capture data (such as getting an email), and the process may continue by adding a line that follows the start events. Many start events have a middle icon that indicates the event’s trigger. A start event with an envelope icon, for example, shows that a message has arrived thus initiating the process.
End Event	Finally, a single thick black line is used to style the end events. The end events are always thrown since there is no process to catch after the final event.
Timer	Upon passing a predetermined number of milliseconds, a single timer event happens once. Every time a predetermined amount of milliseconds has passed, a periodic timer event takes place. An event delay is the period between periodic events.
Gateway	Gateways are a BPMN notation for controlling the flow of a process. A gateway is a decision point connected by sequence flows that determines which (outgoing) sequence flow to take based on the evaluation of the condition(s) supplied. Only sequence flow affects the flow of activities, according to the Business Process Model and Notation (BPMN) standard, but message flow does not.
Task	An activity is a broad phrase for a corporation’s ’work’ done as part of a process. Various activities are critical components of BPMN because they generate business processes. An activity might be atomic (a task) or non–atomic (compound) in nature (a sub-process). Both standard processes and choreographies use activities.

#### Flow chart

A flow chart represents the flow of a process or workflow in the form of sequential steps. It is used to process the model [11]. Therefore, in its representation, it can only represent the process. Moreover, varieties of symbols are provided for the presentation of the start of an activity, input and outputs, and processing, and an ending symbol is provided. Due to less presence of the notations, it is easy to modify, and new things can be added to it easily. It is easy to use as only a few notations are available. For users, its understandability is equally good. However, it cannot be used for presenting in simulation or making it as a simulation as most of the notations required for simulation are missing.

#### PetriNets

It is a graphical, mathematical model in which the process is shown as a graph. In it, arcs are used to connect the transitions and places. Its representation is minimal, making it difficult for the users to understand; thus, inexperienced users find it challenging to understand its functionality. Due to few elements for the representation and underlying logical techniques for making the diagrams, a high level of experience is required for modeling complex systems. Some tools are available for supporting it in simulation, but they cannot represent a business process, making it challenging to use [12].

#### Data flow diagram

Show functionality of the system with the help of function and data flow. It is a powerful technique for designing a business process. It represents the inclusive view of process and activities, and concerned information movement is depicted by the flow of elements like processes, data stores, terminators. Due to functional decomposition, the easily a new process can be added. Due to the availability of simple elements, it is easy to use. Easy for inexperienced users too. Both conceptually and presentations are easy to use as well as understandable. More detailed diagrams are available. DFD is not supported as a simulation for modeling business processes [13, 14].

#### Role activity diagram

Roles are represented within processes and their interaction is represented. The focus is on users, resources, activities, roles, status, and interactions between system participants. In this type of method, the focus remains on the role. Explicit representation of roles and events results in the unsupportiveness of services. As in the model, the focus is role and role represent or work on different tasks. Analysts can refine and amend certain activities by disturbing the whole model with the aid of defined roles [15]. There is a set of easy-to-use different symbols available. Flexibility in representation makes it easy to use and build large systems. It is easy to understand as it gives the whole picture of the system, so it provides a greater aid in designing extensive system process simulations. As it supports working with business process [16]. It supports simulation, as it gives a complete picture of the system as told before.

#### Business use-cases

It stands for a process that develops the modern object-oriented development approach. Natural language’s representation of it is ambiguous and inconsistent. The name, aim, prerequisites, trigger, and fundamental and alternative procedures are the main considerations while developing use-cases. The business object-interaction diagram’s flows are supplied in written form rather than a graphical representation. The actor’s amenities are its primary concern. Text is used to represent the actors’ effort. Because it is shown as text, it is simple to modify. As a result, updating or adding new information is simple. Because it is a textual representation, it is simple to utilize. Writing the use-cases does not require any special knowledge. As a result, it is simple to use. Considering that the depiction is in words, it is simple to understand. It couldn’t be used as a simulation because of its text representation [13, 17].

#### Business process modelling notation

This notation is used to represent the business process. It is a richer representation of the business process. It has flow objects, connecting objects, processes, artifacts, gateways for decision making. It supports all activities in a business process. It is considered a powerful tool for representing business processes. Due to its well-structured organization, a business process can be represented easily more than that improvement can be done easily. Easily used and readily understandable by both business and technology users. It was well defined so that inexperienced users could easily use it. The main purpose is for the user to understand the whole business process easily. Its simple representation makes it easy to understand by the business analyst as well as the technical users. The ability to test processes and visualize them before implementation s supports simulation [18, 19]. Different simulation tools like Simul8, BPSimulator, etc. allow importing BPMN to use as a simulation. Different notations are used to design the BPMN model. Table 1 shows the basic notations that are used in the converter.

Above discussed techniques shows their usage in modeling any process and these are the commonly used techniques. Their usage in making simulation model is main concern here. Table 2 discuss and summarize all the techniques in terms of their flexibility, usability, understandability, and their use to build the simulation models. From the table it is clear that almost most techniques are flexible means they are easy to change or modify but petri net and role activity diagrams are difficult to modify. Similarly, usability is difficult for petri net while for all other techniques it is easy to use. Another attribute is understandability, for petri nets experience is required to work on it. The last feature discuss simulating feature for the techniques. For flow-chart due to limitation of the modeling notation its simulation tools are not available. Petri net simulation tools are available like JPetriNet, Petri.NET Simulator etc [20]. For DFD and role activity methods no simulation tool available as their focus is only on data and roles respectively. The BPMN is widely used modeling tool for showing business process. There are multiple tools available in which BPMN simulation can be done like BPSimulator, BIMP Simulation tool etc.

**Table 2:** Summary of different modeling techniques.

Techniques/Methods	Flexibility	Usability	Understandability	Use to build Simulation
Flowchart	Easy to modify	Easy to use	Easy to understand	No tools available for simulating flowchart.
Petri net	Difficult to modify	Difficult to use	Experience required for understanding	Petri net simulation tools are available but it requires understanding experience
DFD (Data Flow Diagram)	Easy to modify	Easy to use	Easy to understand	No tools available for simulating DFD.
Role activity diagram	Difficult to modify	Easy to use	Easy to understand	No tools available for simulating role activity diagram.
BPMN	Easy to modify	Easy to use	Easy to understand	Different tools available for simulating BPMN. More than that simple BPMN diagram can be converted to simulation model.
Business use cases	Easy to modify	Easy to use	Easy to understand	It is represented as text so it cannot be simulated.

### Business Process Re-Engineering (BPR) and simulation

BPR has changed over time, from the standpoint of various processes to the technology elements related to processes. BPR is seen as an antiquated method for re-engineering corporate processes. The ability to reinvent has mostly depended on management insight, inventiveness, and common sense in managing change. As a result, by definition, BPR is in favor of dismantling the entire business process and replacing it with brand-new ones. Creating definitions is simple, but putting them into practice is another matter. On the other hand, since definitions have changed, studies appear to now include technical elements to enable the reengineering of business processes [21].

**Fig 1:**
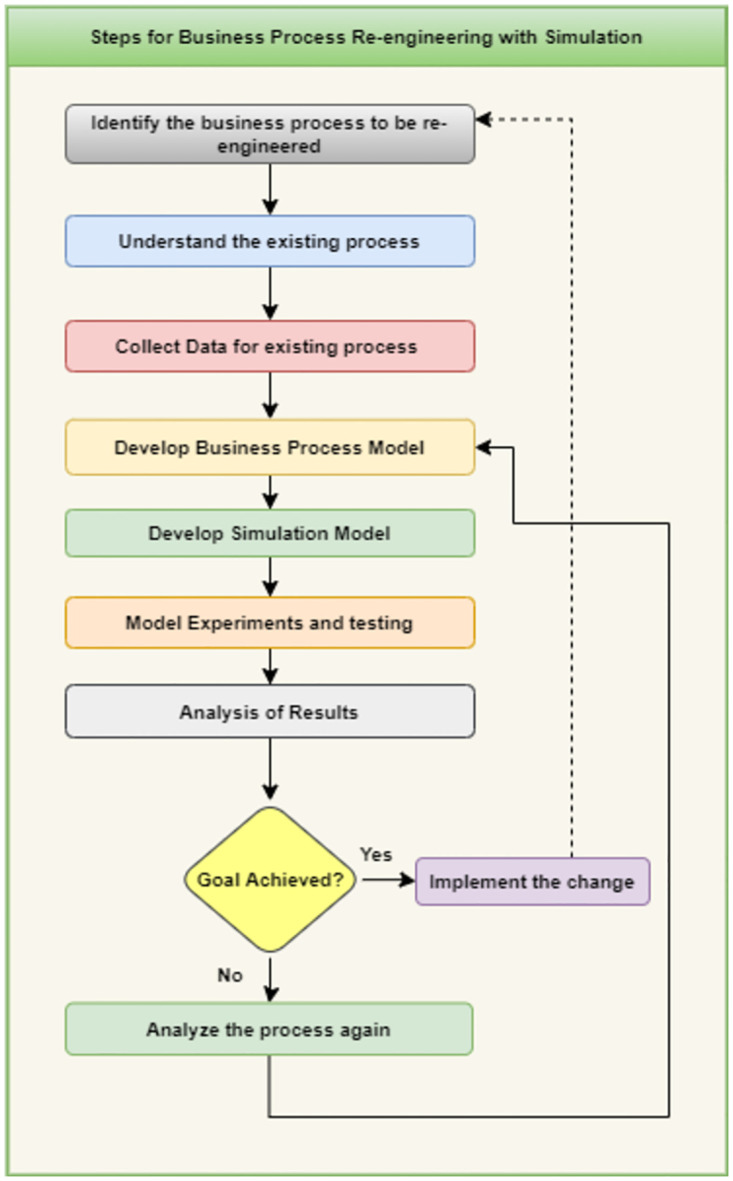
Steps for business process re-engineering with simulation.

Fig 1 shows the steps for business process reengineering by using simulation to get the verification and validation of the change either it is useful or not. The description on each step is as follows,

*Identify business process to be re-engineer*: In order to start the BPR the changes that are required in business process are identified. It starts by identifying external issues, reviewing the current procedure, and comparing it to best practices from other implementation in the same area. Specific business goals are identified, such as improving output quality and reducing costs and time, improving any delayed process etc.
*Understanding existing business process*: Having complete awareness of the current business process is also very important. The business analysts will benefit from this information as they make modifications and pinpoint process flaws. The limitations and degree of adjustments must also be set in order to create simulations with an appropriate model.
*Collect data for existing process*: The gathering and analysis of pertinent real-world business data is the focus of this phase. Data collection provides a quantifiable standard to evaluate the benefits and drawbacks of process improvements. If there are any abnormalities at this point, a loopback can be used to explain the modelling goals.
*Develop business process model*: Business process models are used to make it easier to grasp "how" a process works right now and "what" it does. After then, steps are done to enhance the process. Expertise and inventiveness in simulation modelling are most evident at the model-building stage. The creation of process models requires many resources, though. The goal is to comprehend the issues, identify the information flow limits, and look for the best solutions to enhance the system’s overall performance. This model will be then used by the business analysts for studying the system. In addition, it is the static view of the process. BPMN is widely used as a modeling technique for developing business process as a graphical representation.
*Develop simulation model*: After modeling the business process the analytical and simulation procedures follow. It shows the dynamic behavior of the business process. To capture a dynamic image of models using simulation software, Simul8, Arena, and Anylogic are a few simulation tools available. As results of the simulation model help the business analysts to get the clear picture of the change prior to the implementation. Therefore, it is helpful phase during the process of BPR. Developing simulation model is also important at this stage because of many reasons like many business processes are unpredictable and contain random variables. Other than this the primary components of the business process, activities and resources, interact. Moreover, organizational business processes are impacted by one another and altered by parties outside the organization.
*Model Testing and Experiments*: Model testing and experimentation is another important phase of the BPR with simulations. The model testing help to analyze business processes in the following terms:
  - Identifying waste of resources and bottlenecks at any stage of process.
  - Process reviews are scheduled in order to boost performance.
  - Choosing procedures with better designs will yield greater results.
  - Cost assessment.
  - Evaluating the effectiveness of novel processes.
Experimentation provides accurate and trustworthy estimates of the outcomes. The model is run numerous times throughout the experimental phase, and important statistical tests are carried out to determine the steady state and run length of the model.
*Analysis of Results*: To determine whether the outcome obtained has met the intended outcome, the output from the model experimentation step is examined. A key component of process evaluation is the identification and measurement of performance indicators. If performance indicators are unsatisfactory, it means that earlier stages (such as experimentation, testing, or data collecting and analysis) need to be reevaluated before moving on to the recommendation phase. As a result, this adaptive system corrects early flaws.
*Analysis of Goals*: The last step in the process in either the goals successfully achieved or not. If the analysis of results shows positive in favor of the change, the organization is prepared for the reengineering or improvement phase. The objectives that exposed the need for reengineering or improvement are carried out. The new process’ design is a part of this step. Either a method of progressive improvement or a method of drastic change is used, depending on the specific circumstances. Existing processes must continue to operate without interfering with the environment in which new ones are installed until that installation is complete.

If the analysis of results show negative impact of the change then the business analyst may change the process or restart the BPR activities again depending upon how much difference the change is causing.

In [22] the author shows the importance of BRP on enterprise process. The reengineering of the enterprise’s processes, particularly those crucial work processes that directly interact with and add value for customers, forms the basis of business process reengineering. Therefore, they are the company’s main priorities. People are urged by business process reengineering to reconsider the current management process and look for fresh approaches to enhance the enterprise’s management from process.

The key benefit of simulating a business process analysis is that it made it possible to include variables like unpredictability and interdependence in order to get a realistic picture of how the process performed. A range of process performance metrics, including lead-time, resource use, and cost, might be predicted by the simulation. The visual dynamic display also offered a communication venue for validating the model and explaining how the newly planned activities function and how they contribute to the efficiency of the entire process [23]. Renaldi discussed the improvement in the public administration by implementing business process reengineering, verifying, and validating the results by building a simulation model. The author discussed the improved process by combining the BPR and simulation in public administration. The effectiveness of the organization was first evaluated, and then internal processes were redesigned to improve performance using a combination of BPR and simulation [24]. Another BPR and Simulation technique is implemented on call center in another paper. After successfully implementing the change in call center it was mentioned how crucial BPR modelling and analysis are to the process of reengineering. Because feedback control and stepwise performance analysis reduce the risks of reengineering failure by fixing early modelling and simulation issues, a flexible and iterative BPR and Simulation methodology is advised. The initial time, human and financial resources required applying the BPR and Simulation to any business process are recovered in efficiency gains or in the cost averted from mistakes made during technology or process change. The reengineering with the combination of simulation together have many features that can help improve any process [25]. Health care systems are critical systems; any change in the process must be made with real attention [26]. The BPR together with simulation in such critical systems also played important role. In [27] the wait time for emergency service patients is decreased by analyzing the existing process and simulating the change process and getting results. To reduce patient wait times, hospital administration consider making certain adjustments to the emergency care procedure. This study simulates the emergency treatment procedure on a computer and assesses the impact of various alterations on patient wait times. The actual numbers are used to validate the simulation results. After analyzing the results, the author recommends the changes in the process so that the wait time of the patients coming in the emergency department is decreased.

## BPMN and simulation

The Business Process Management Initiative developed and promoted the language known as Business Process Model and Notation, or BPMN, which first appeared in 2004. (BPMI). Eventually, the Object Management Group accepted BPMN as a standard (OMG). The first version of BPMN was developed in order to standardize the graphical depiction of business processes. It provided a set of "visual symbols" for the various process parts that each had a distinct meaning and could depict the multiple possible combinations [28].

BPMN’s primary objective is to offer a notation that is clear to all parties involved in organizational processes, from business analysts who create or define business process models to technical developers who are in charge of developing the IT infrastructure for those processes, and finally to all users who will oversee and manage the developed processes. To make it simple for modelers to identify and differentiate between various elements, the language elements’ notation was graphicalized. For instance, business process operations are typically represented by rectangles, whereas choices are displayed by diamonds [29].

Today, it is commonly accepted that simulation experiments are a trustworthy and reputable source of information regarding how to support organizational decision-making. In fact, managers are better equipped to root their decisions when they have the ability to predict, in a concrete and intelligible way, the likely outcomes of a decision before making it in the actual world. Simply said, simulation helps managers with their decision-making responsibilities by enabling them to create and assess a variety of potentially interesting scenarios. The expenses and hazards associated with evaluating "what if" scenarios in a real environment are removed when "what if" situations are analyzed using simulation.

There are a variety of solutions available, including Bizagi, BIMP, BonitaSoft, BPSim, SAP, and Visual Paradigm, to convert BPMN to functional simulation. To simulate a business process, an analyst had to adapt the model to the specific language of the BP simulation tool they had selected. Such a condition is unjustified and uncomfortable due to the duplication of effort. There are various restrictions when modelling BPMN diagrams other from this. The implementation of BPMN varies depending on the vendor. The analysts must adhere to a unique set of guidelines for each vendor in order for the BP simulation to function. Additionally, the providers offer a few limited functionalities. No tool provides the entire range of capabilities. It is not possible to export or import a working simulation from one tool to another. Each tool must have its own set of conventions for creating BPMN. BPMN simulation tools cannot be used to develop complicated processes due to functional restrictions.

The analysis of a few BPM tools [30] that forms the basis of this research has shown that, in addition to a minimal set of features that are required to do straightforward simulation work and are shared by all of them, there are considerably different simulation capabilities among tools. On the one hand, several BPM tools can already be used for significant simulation work, while on the other hand, there are still some that lack crucial simulation features.

Whatever the case, it is evident that BPM tool developers, particularly those who support the BPMN standard, are already making an effort to include simulation features in their products. It is reasonable to anticipate that in a few years, simulation elements will be included as standard in BPM tools.

## Reverse engineering

Reverse engineering, often known as back engineering, is the practice of disassembling software, equipment, aircraft, architectural structures, and other products in order to learn how they were made. Smaller pieces of larger products are regularly disassembled as part of reverse engineering. By applying the reverse engineering method to discover how an item was made, you can recreate it [7]. Different researchers are working on the transformation of one technique to other using the concept of reverse engineering. In [31] The gap between using the BPMN model and the UML is bridged by a tool that combines the advantages of model transformations and traceability, ensuring and maintaining alignment between business modelling and requirement elicitation. They created rules for bidirectional translation between use case and BPMN models and suggested an interim integrated model for case models. Reverse engineering is crucial in this process to comprehend the transition. In another approach, the author describes the enterprise process reverse engineering (EPRE) method, which may be used to analyze business processes and assist with tasks including process change. The method offers designers and users direction for process redesign as well as for creating the present process model by evaluating common business forms [32].

## Approach

Business analysts can examine changes to existing processes or the introduction of new processes with the use of simulation and business process reengineering. The simulation and business process re-engineering are depicted in Fig 1. Our work is the reverse process of the two steps to create BPMN and then simulation while we worked on simulation first and then generate BPMN from it. To determine whether the modification or new procedure is feasible in this case, we will first create a simulation. Our framework will auto generate the BPMN model for the specified business process once the simulation model is complete. It will help business analysts to save time and resources in making BPMN which they will get auto generated on every change in simulation model.

This section discusses the methodology proposed in converting the simulation model to BPMN. This tool is build-using Java; a desktop application with backend database is developed in SQL server. For testing, the model Simul8 and BPSimulator is used. Simul8 is simulation-building tool widely used in industry. Using this, one can build discrete event simulation. BPSimulator is BPMN simulation tool that converts BPMN to simulating BP Model. The developed framework is then tested with case studies, after which the results are analyzed.

### Framework

This research aims to make a framework that converts the simulation to the best corresponding BPMN after mapping the corresponding notations. This work intends to use reverse engineering to understand the simulation build from the BPMN and then reengineering to build BPMN from the simulation model. The framework will also generate an XML that contains three parts humans, products, and transactions present in the model. This XML can be used in creating the database based on the identified entities and their attributes.

**Fig 2:**
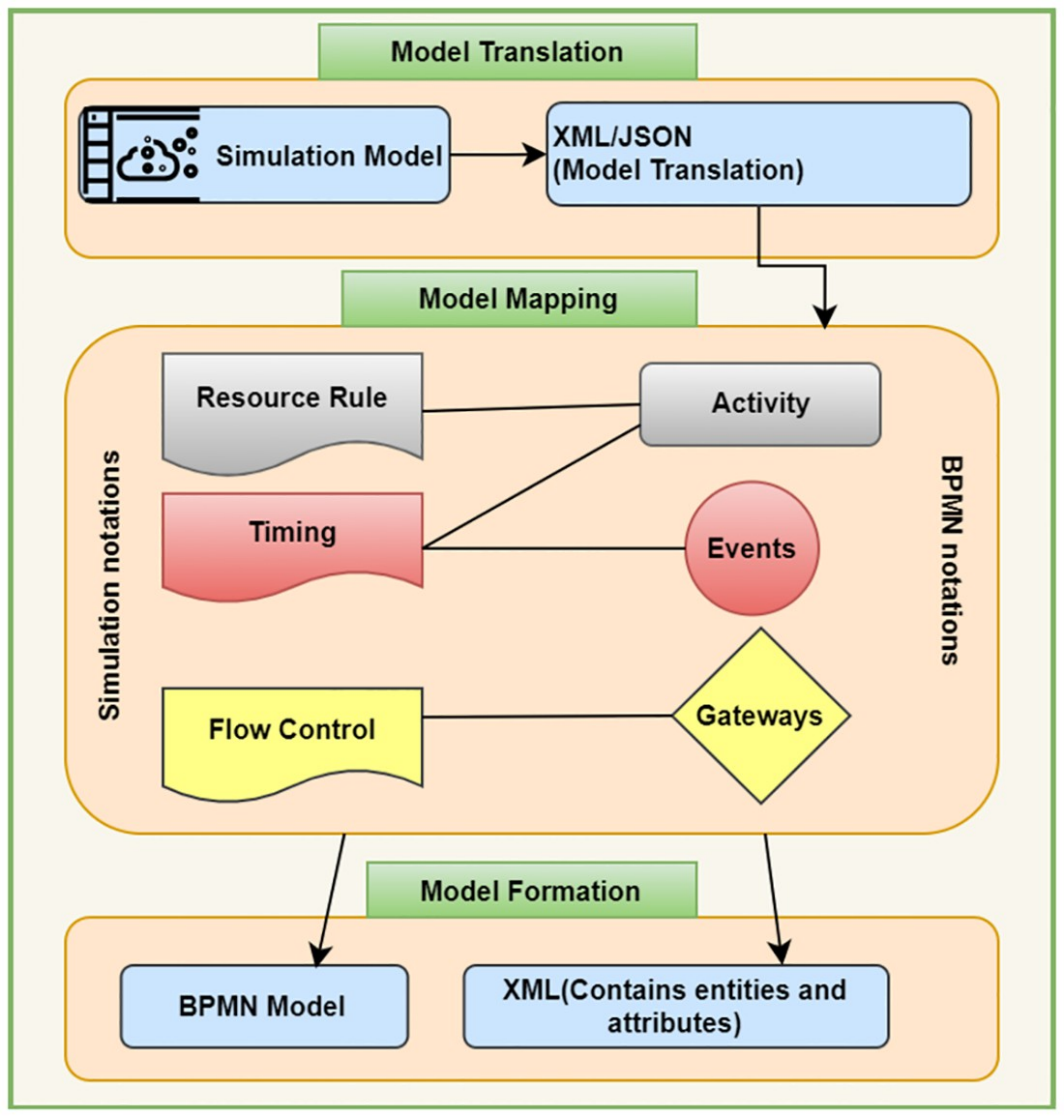
Generic framework showing three layers, model translation, model mapping and model formation.

This framework is divided into three layers, as described in Fig 2, model translation, model mapping, and model formalization.

**Fig 3:**
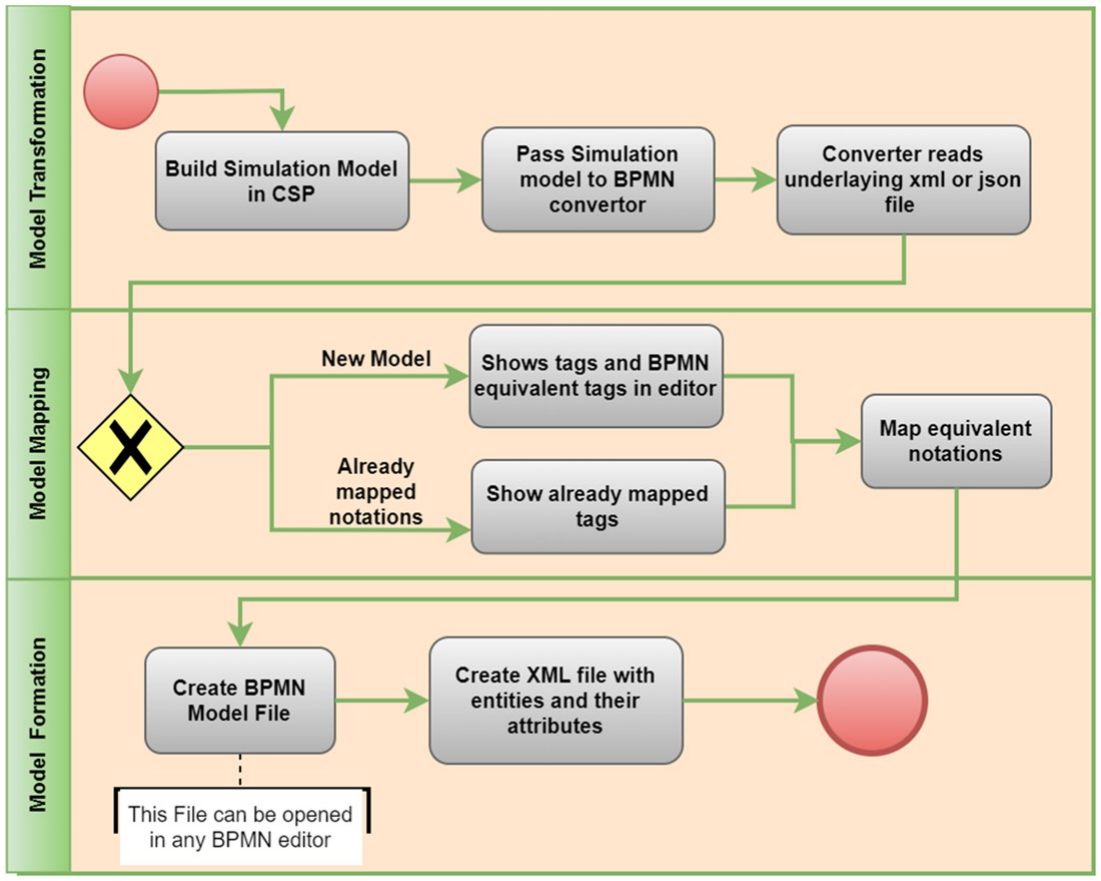
BPMN model showing the detail steps for all three steps. Model Transformation, Model mapping and Model formation.

The generic framework shows the model translation, model mapping and model formation as three layers of the architecture. Fig 3 shows the BPMN model to help understanding the flow of working between these three layers. The working of each layer is described below.

#### Model translation

The first layer is the input layer, and it contains the simulation file. The simulation file is model file build in simulation tool. The simulation model file contains the dynamic view of the business process. The simulation models has backend developed either in XML or in JSON. At this layer, the simulation model is given as input to the tool. The backend XML or JSON file is then read. The XML or JSON files contain the tags from the simulation model file that will be used for mapping in the next stage.

#### Model mapping

The second layer is the mapping layer that maps the simulation model notations to the BPMN model. It first checks for the new modelling tags from the XML or JSON. Then display the tags with corresponding BPMN notations. After mapping successfully the required notation the data is then saved in database for further use if the model from the same simulation tool is given as input again. All simulation models have some generic terms used for understanding the simulation. Some that are used in this framework are as follows.

- *Resource*: A service or a tool needed to perform the function is called a resource.
- *Timing*: This notation refers to the time required to complete a function. It can be a waiting queue.
- *Flow Control*: After performing a process, the way or the navigation to the following process is defined by flow control. Multiple processes can be performed after a single process, or a process can only be performed after completing one or multiple functions.

The above notations are mapped with the notation in BPMN. The notations covered in this study are as follows:

- *Activity*: An activity is a broad phrase for a corporation’s ’work’ done as part of a process. Various activities are critical components of BPMN because they generate business processes. An activity might be atomic (a task) or non–atomic (compound) in nature (a sub-process). Both standard processes and choreographies use activities.
- *Event*: Something that occurs during a process is referred to as an event. Start, middle, and finish events are the three main events in business process modeling. The three event types are sometimes known as catching events (that respond to a trigger) or throwing events (that do not react to a stimulus), which the process triggers.
- *Start events*: Each process must begin with a starting event. All start events capture data (such as getting an email), and the process may continue by adding a line that follows the start events. Many start events have a middle icon that indicates the event’s trigger. A start event with an envelope icon, for example, shows that a message has arrived thus initiating the process.
- *End events*: Finally, a single thick black line is used to style the end events. The end events are always thrown since there is no process to catch after the final event.
- *Gateways*: Gateways are a BPMN notation for controlling the flow of a process. A gateway is a decision point connected by sequence flows that determines which (outgoing) sequence flow to take based on the evaluation of the condition(s) supplied. Only sequence flow affects the flow of activities, according to the Business Process Model and Notation (BPMN) standard, but message flow does not.

#### Model formation

The last layer is the model formation or the final output. It contains the result as a BPMN model file that can be opened as a BPMN 2.0. For opening the file Eclispe can be used. More than this BPMN model file another XML is also generated. The XML file that contains the entities that are in simulation model and their attributes. Table 3 shows the list of functions that will be used to generate BPMN from the XML.

**Table 3:** List of java function with its description.

Functions	Description
SimulationFileReader	SimulationFileReader gets the input as a simulation file and read the notations of the simulation such as generators, queues, checkpoints, functions etc.
readJSONFile	JSON is a file format generated as backend simulation model. This function reads the JSON file of the simulation model and read the notations of the JSON file. After that, it display the detected notations in a grid.
readXMLFile	Some simulation models have XML as their backend file. This function reads XML file and separate XML tags from this file. After successful attempt, it shows the tags in grid.
BPMNGenerator	BPMNGenerator function inputs the file of the BPMN Diagram, which have all the mapped notations of BPMN and generate diagram according to the file.
XMLFileWriter	XMLFileWriter gets the process and write a file a file that have specific XML format. It writes the notations of the simulation model that are extracting in file reading now its turn to gather all the notations in a file so that we can map then with BPMN.
generateXML	When the notations are gathered in a file then we will generate XML file by using generateXML so that a file is generated which contains the notations of the simulation model of both tools.
BPMNFileWriter	When we map the notations, we map one by one notation of the simulation and map them with BPMN diagram notations. We write all the mapped notations in a file by using BPMNFileWriter function.
generateBPMN	When all the notations are mapped and write in a file then we generate BPMN by using the BPMN file that contains all mapped notations of BPMN.

For converting the simulation model to the equivalent BPMN diagram we developed the framework by using Java in Eclipse IDE. At the initial stage, the function SimulationFileReader() is used for accepting the simulation file and reading the notations as we have worked on two different format inputs one is XML and other one is JSON. Both have their separate format that why the readXMLFile() function is used for reading models that generate XML schema as its backend file and ReadJSONFile() for reading the notations of models that generate JSON file as its backend file. When converter reads the files which contain the notations of the simulation model now it will write these notations in a file as well as to the database by using the function WriteXMLFile() and generate the XML file by using function generateXML(). Now simulation file is ready to map and it can map those notations with BPMN diagram notations. After successful mapping of the notation now it will write a BPMN file in which it will write the mapped notations for this function named BPMNFileWriter() will be used. Finally, the BPMN notations are mapped now in a file now it will use generateBPMN() for generating the BPMN diagram. The generated BPMN file can be opened and viewed in Eclipse as well as any tool that support BPMN 2.0.

### Simul8 to BPMN Architecture

Simul8 is a CSP that provides users with an intuitive visual modelling interface to quickly build precise, adaptable, and resilient simulations. Table 4 shows different notations used in Simul8 and its meaning in the simulation model.

**Table 4:** Simul8 notation description.

Simul8 Notation	Description
Start point	To start a simulation process in simul8 it is the first point. This is the basic entry point for the simulation to start. More than one start points can be added to the model. They run their adjacent logics independently. Each start point can work on different statistical distributions.
Queue	Queues are building blocks in simulation model. Here an entity will wait until the activity or the resource is available to continue work in the model.
Activity	An activity is a location where work on work items is done. Working at Activities typically takes time, and occasionally resources must be available. A Work Item may undergo some sort of transformation at an Activity (perhaps by changing one or more of its labels). Depending on the routing rules that can be provided, the Work Item after completion of the work may be forwarded to another simulation object or to any number of distinct simulation objects.
End	End is used to show the simulation ends. When the work in model completes it is used to end the process and here the results are collected. The result manager is used to collect results.
Resource	Resources are things that must be present in the simulation for activity to work on a Work Item. These activities cannot begin operations until both a Work Item and the required Resources are on hand. Examples of resources are machine operator, labor etc.
Routing Arrows	To connect all the elements of the simulation model the routing arrows are used.

**Fig 4:**
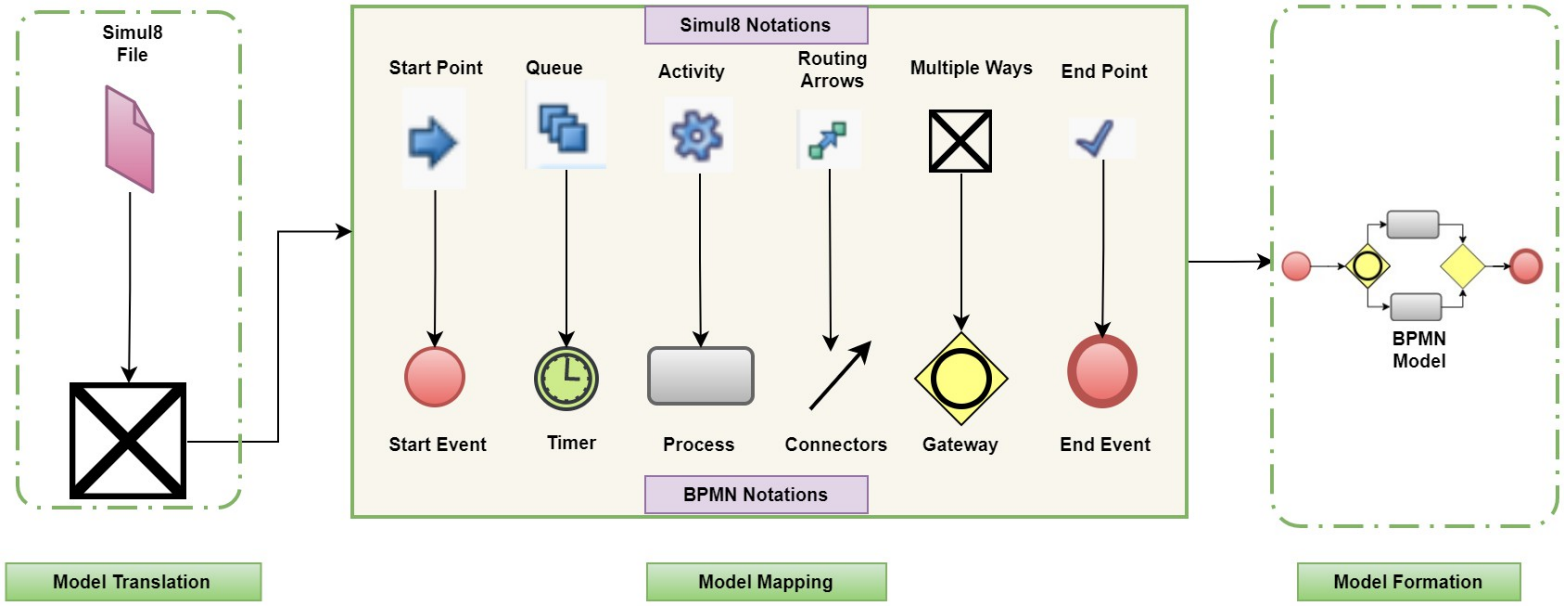
Simul8 to BPMN convertor takes Simul8 model file, maps the notation to BPMN and generates final model.

Fig 4 shows that the simul8 conversion from the model to BPMN needs to map the notations modeled as a simulation. The conversion starts from the model file, where it is converted to the corresponding XML file. Afterward, it maps to the BPMN in the same manner as the BPMN is mapped to the simulation model. The conversion takes place in reverse to get the required BPMN working file from the model for which the business model is not present.

The simulation model file is converted into the XML file that contains the information about the model and its notation. This notation is in the following form, where it is mapped against these processes in the format. After successfully reading the file and mapping the file, the framework will generate the BPMN file. This reengineering work is illustrated in a simple model as shown in Fig 4 about the simulation. The making of juice process is shown in this simple model. Here, the simulation begins with the start point, converted to the start event of the BPMN, as shown in Fig 4. Similarly, the queue shows the delay in the process, and against this process, the intermediate event is mapped as the reverse process. After that, there is an option between making juice, cutting it, or mixing it. It is then being converted into the corresponding inclusive or gate in the BPMN. Once the process is done, the endpoint will end the simulation process, and in the corresponding BPMN, the end event will be generated. The routing arrows are used as connectors in BPMN to connect the whole model.

### BPSimulator to BPMN Architecture

BPSimulator is also a simulation software that simulates the given BPMN. In this architecture, the notations are mapped in the reverse process. As the BPSimulator generates the simulation file in JSON format. For the conversion, the JSON formatted file has to be read first to understand the simulation files, extract the notations of the simulation model into a file, and finally save the file. After reading the simulation notations, they should be mapped to the BPMN. Table 5 describe the building blocks of simulation model in BPSimulator. Main notations include function, task generator, event, checkpoint and resource. The description about these notations and their usage are also shown in this table.

**Table 5:** BPSimulator notations with description.

BPSimulator	Description
Function	As name indicates, it is used to represent the action that occurs during the process.
Task generator	It is the start point for the process. Tasks are starting from this point in the process.
Event	This represents an intermediate event that shows an intangible outcome of a function.
Checkpoint	A supporting component for controlling task flow and monitoring process parameters at various stages of execution. End of the process or completion of some activity is usually represented by this component.
Resource	Any service or resource that is required to perform some activity is shown by resource.

**Fig 5:**
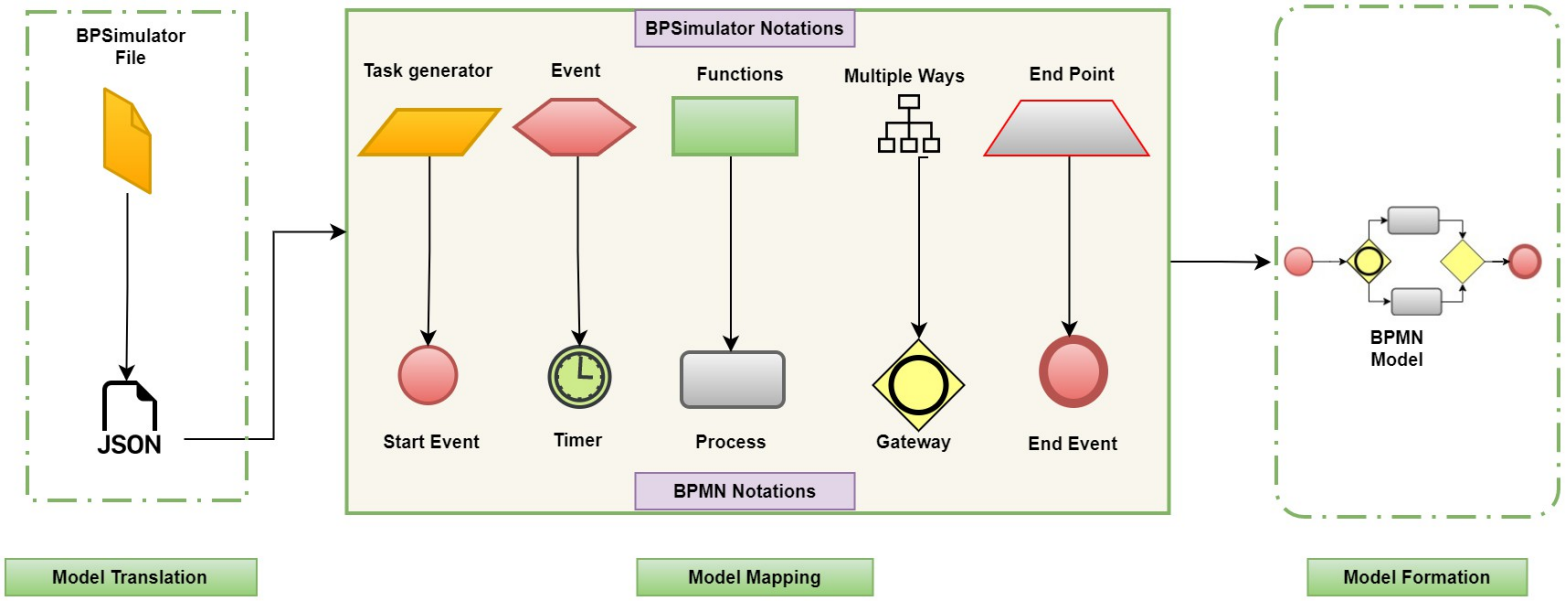
BPSimulator to BPMN convertor takes BPSimulator model file, maps the notation to BPMN and generates final model.

The generic architecture is translated to use BPSimulator as input simulation model. Fig 5 shows the system’s behavior as the BPSimulator files enter, generating an XML file of the simulation model with all the notations of the simulation model. Subsequently, the notations are mapped; the upper layer notations are from the simulation model, and the lower layer notations are the BPMN notations. The notations of simulation generated are converted into a start event in BPMN. For the events, there are the timers, as the multiple ways are converted into gateways, all the functions converted into processes, and the checkpoints change into end events. When all events processes and flows are mapped with BPMN diagram notations, it is clear that the BPMN notations are written into an XML file after mapping. The generated file is called a BPMN file in XML format and can be used in generating the BPMN Diagrams.

When talking about the mapping in the BPSimulator tool there are notations just like task generator, which starts the simulation and converts it into the BPMN notation of start event. The checkpoints in the simulation are converted into the end event of the BPMN. When the queues of the tasks appear in the simulation, there is a notation of an event in the simulation, which is mapped with the intermediate timer event of the BPMN. Every process, just like cutting, stitching, and printing, is a business process. The notation of functions in the simulation is mapped with the process notation of the BPMN. When there are sub-processes to complete a process, they are presented with multiple tasks and mapped with the gateways of the BPMN. When the process starts, an open gateway is used, while when it finishes, closed gateways are used.

## Case studies implementation

This section explains three case studies. The models of all these are built-in Simul8 and BPSimulator. Subsequently, these models are passed to the framework, which translates them into XML. Afterward, the XML files are mapped into the corresponding BPMN file.

### Bank model

In this part, a bank-working model for the customers, where they can take services of the bank in the form of using the ATM service, visiting the advisor, or the teller is built. When customers enter into a bank, they have choices to perform tasks in it such as using ATM, visiting an advisor, or seeing the teller. Bank advisors give customers advisory information like payment of bills, loan information, depositing money, withdrawing money, foreign money exchange, and bank draft. Tellers help the customer in the routine tasks of the bank related to day-to-day work. It includes making deposits to the account, withdrawing money, or helping customers to issue money orders or cheques.

On entering the bank, a customer has three choices either to withdraw money from ATM or to visit an advisor or the teller. Each task has separate queues in which the customer can wait in case of the ATM service is unavailable or the advisor/teller is busy with other customers. When the services get available customers can access them by visiting the ATM, teller, or advisor. After performing the specific task, the customer can exit the Bank as each task has its separate end. This aspect explains how the customers in the bank are managed.

**Fig 6:**
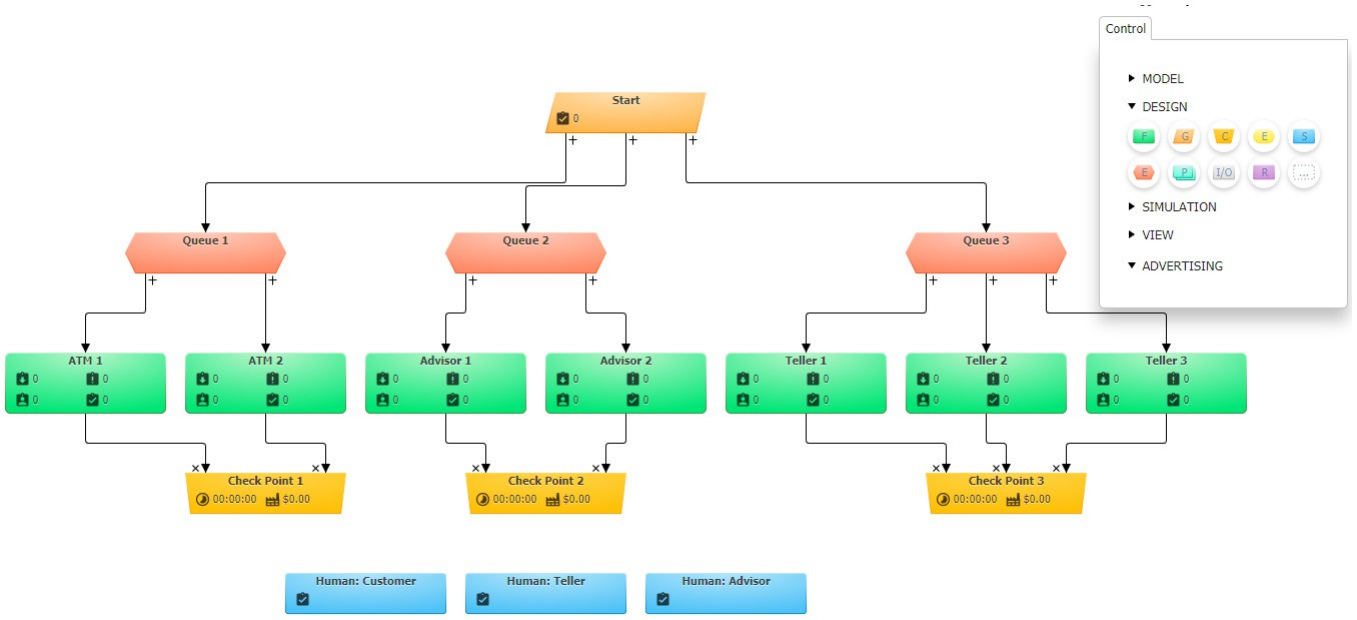
BPSimulator model showing bank activities.

**Fig 7:**
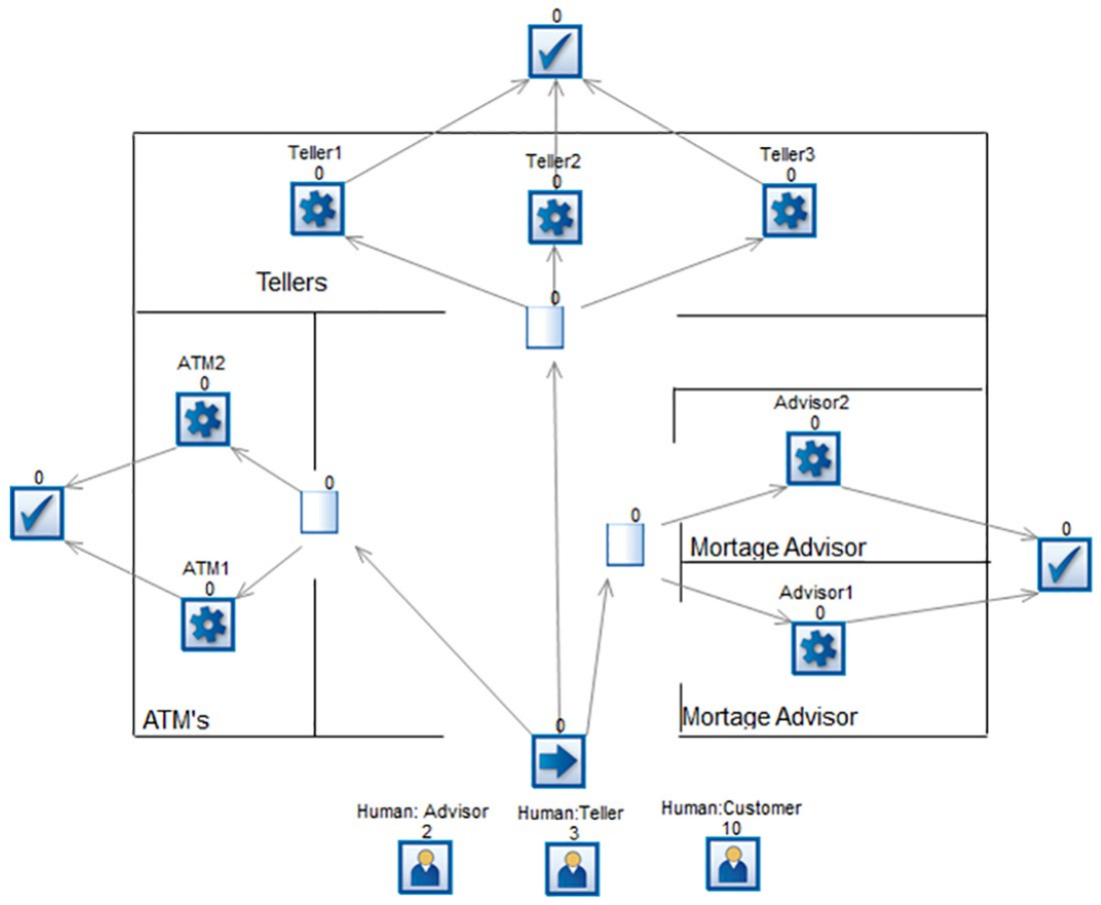
Simul8 model showing bank activities.

**Fig 8:**
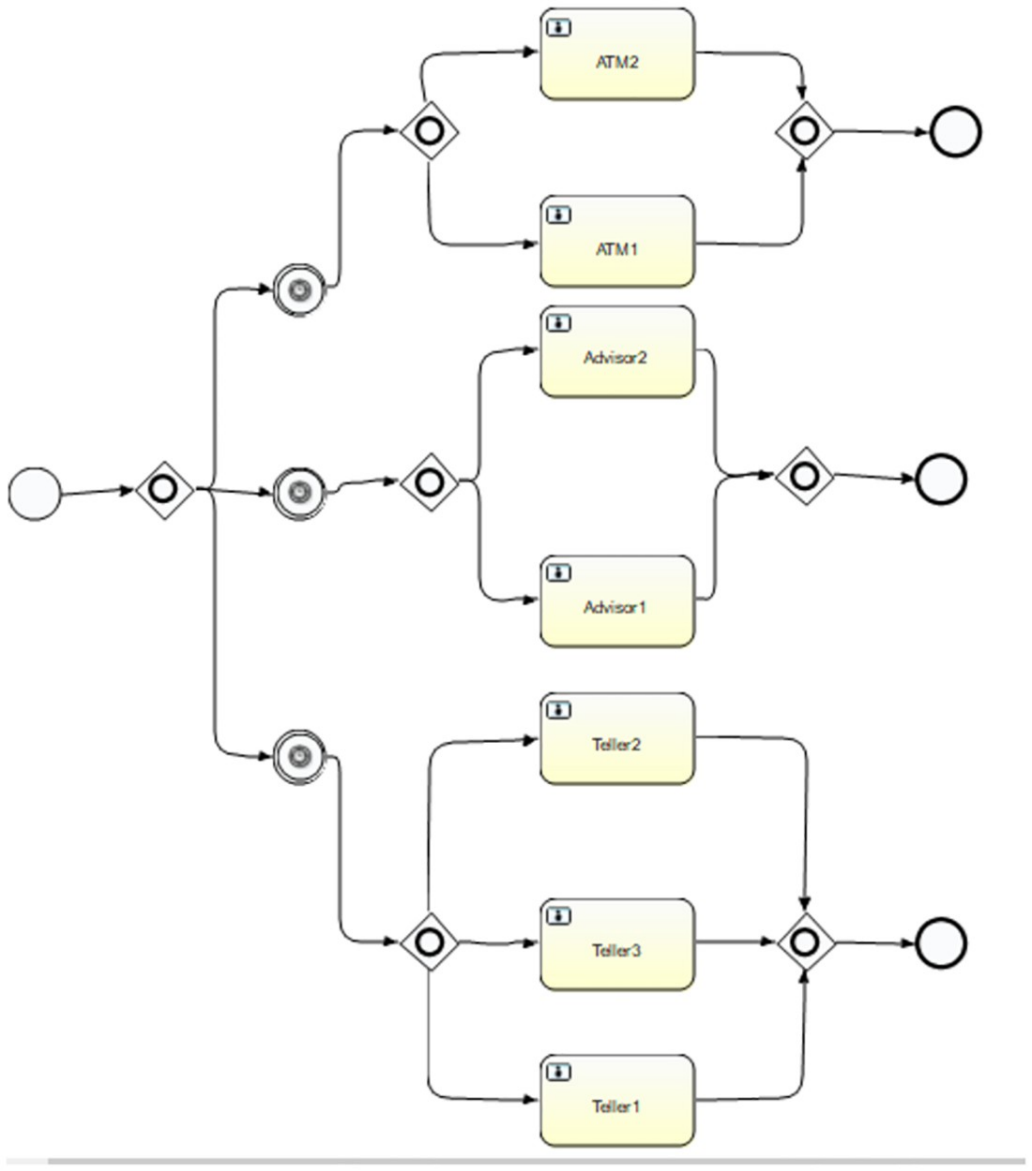
After reading models the BPMN generated as above, for both Simul8 and BPsimulator same BPMN model is generated.

Models have been developed for the bank keeping in view the process of working in both Simul8 and BPSimulator. Fig 6 shows the model generated in BPSimulator. This model uses functions, checkpoint, events and task generator to show the model of Bank. Fig 7 show the models developed in Simul8. This model uses all its notations like start, end event, activity, queue and connectors. These two developed models are then passed to the framework where the XML is extracted in case of Simul8 and JSON is read when BPSimulator is passed as an input model file to the converter. The mapping takes place as described in architecture of Simul8 and BPSimulator, and the final product BPMN model is generated from the given model as shown in Fig 8. Both Simul8 and BPSimulator models produce the same BPMN, showing the process of the bank as described in the model.

### Shirt manufacturing model

Manufacturing industries play an important role in the progress of any country. They involve the production of anything from raw material to the final product. The next model shows the manufacturing of a shirt. It includes many steps like preparing the order, collection of raw material, cutting process of the shirt, printing, and stitching at the end checking the quality of the shirt with the quality tests.

In a shirt manufacturing simulation, a process starts, and then the order will be received in prepare production stage. The first stage is preparing the order that includes the number of shirts being prepared, the design of the shirt, and the preparation of the next steps during this process. In the simulation model, it is shown as an activity of preparing the order. When the order is prepared after this, the steps of shirt manufacturing start. The first step is to collect raw materials for the manufacturing of a shirt. The activity in the simulation model also represents this step of collecting the raw material. Once the raw material is collected, it gets to the next step of cutting the shirts. After cutting, the manufacturing moves towards two steps: a printing shirt and a stitching shirt. Printing of shirts involves making the prints or designs of the shirts, and with this, it moves to the stitching. When both steps are completed, the stitching stage is completed. The last step in the process is checking the quality of the product, which is the shirt. At the last quality of the shirts will be checked. Quality-checking is the last stage of the shirt manufacturing process. After completion of all steps, the end process shows the process end.

**Fig 9:**
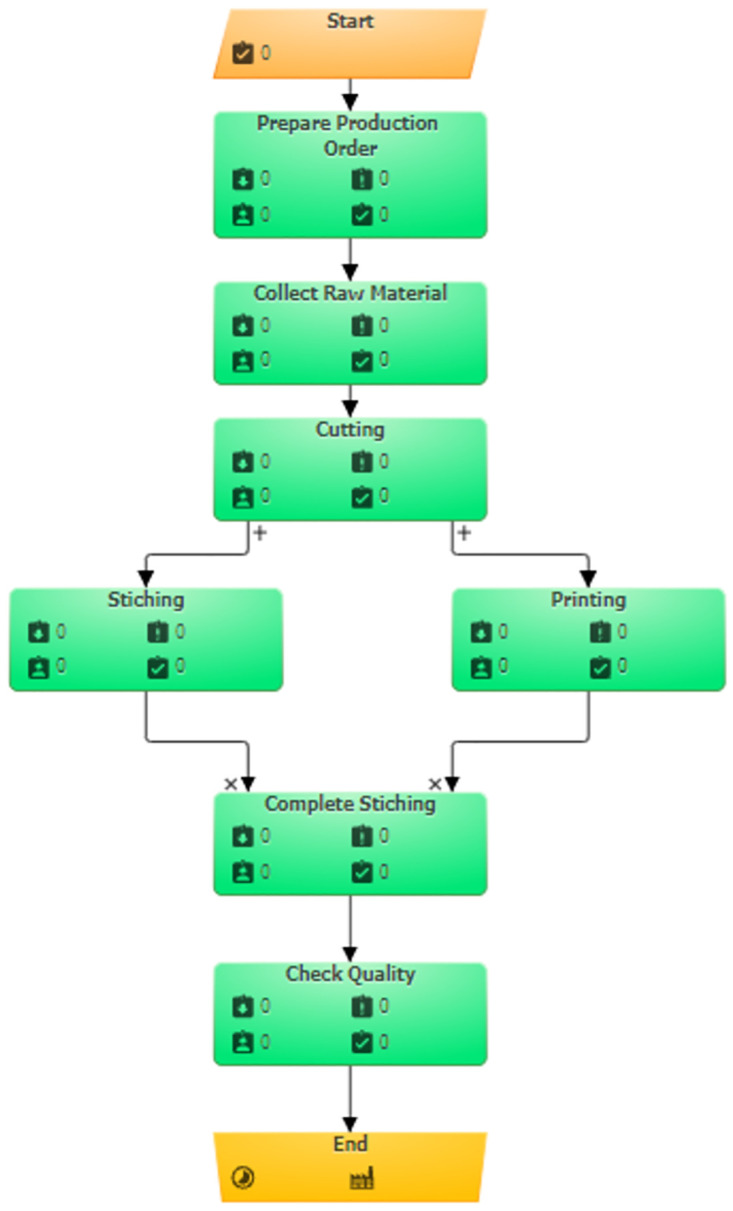
BPSimulator model for shirt manufacturing.

**Fig 10:**
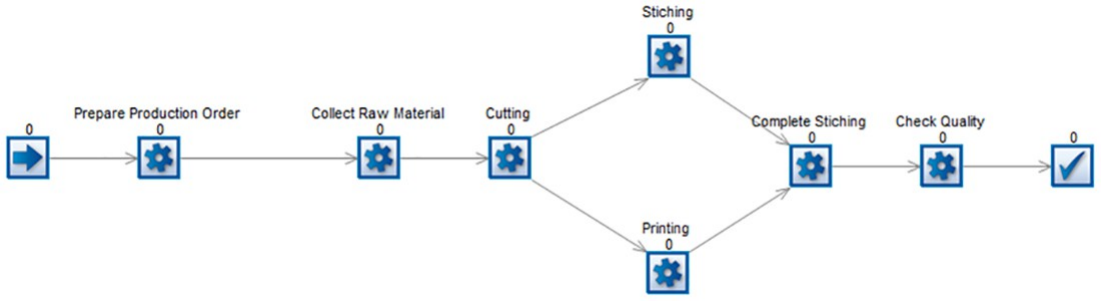
Simul8 Model for Shirt Manufacturing.

**Fig 11:**
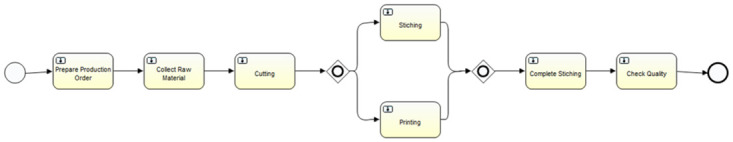
Generated BPMN model for shirt manufacturing, for both Simul8 and BPSimulator model generated BPMN is same.

This manufacturing process is built up as a simulation model in both the Simul8 and BPSimultor. The model build in BPsimulator is shown in Fig 9. On the other hand, Fig 10 shows Simul8 model of shirt manufacturing. Both these models are passed to the converter one by one. After the input of the simulation, models mapping takes place, which results in the BPMN model shown in Fig 11.

## Discussion

The framework developed to convert the simulation model to BPMN. The developed converter is tested on two case studies. One is the bank model in which the customer can either use ATM, visit an advisor for information, or visit the teller for the day-to-day transaction. This model is designed in both Simul8 and BPSimulator. The reason for choosing two models is, to test the converter for both XML and JSON input. Simul8 model generate XML as its backend file while BPSimulator model generates JSON as backend file. While making a simulation of bank model in Simul8, the notations used in this model includes start event, activity, queue, multiple ways, connectors, and end event. Similarly for BPSimulator bank model all the notations described in the architecture are used for testing it against all the notation of BPMN. All these notations are used in the model to verify the conversion of all notations to the BPMN. Table 6 shows the number of Simul8 and BPSimulator notations used in bank model are 14. To show the bank model the simulation models used 14 notation by both of the tools. After converting to BPMN the number increased to 21 in case of bank model. If we look the shirt-manufacturing model there are 9 notations used in both BPSimulator and Simul8. In the shirt-manufacturing model the converted notations are 11.

**Table 6:** Number of notations used to build both models and generated BPMN model.

	Bank Model	Shirt manufacturing Model
**Number of BPSimulator notations used**	14	9
**Number of Simul8 notations used**	14	9
**Number of mapped BPMN notations**	21	11

**Fig 12:**
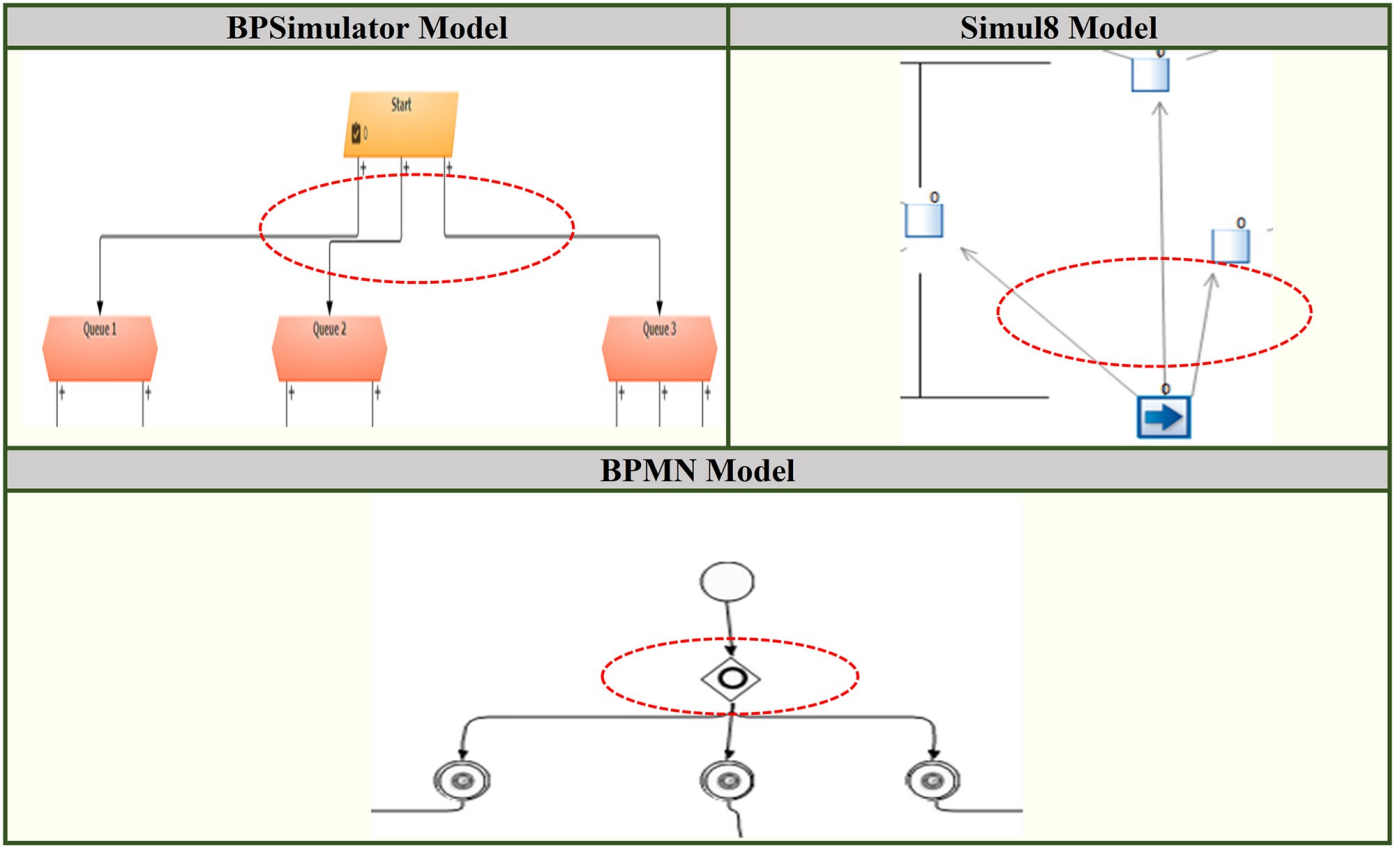
A part of bank model shown in dotted oval in BPSimulator and Simul8 is converted to parallel gateway in BPMN model.

**Fig 13:**
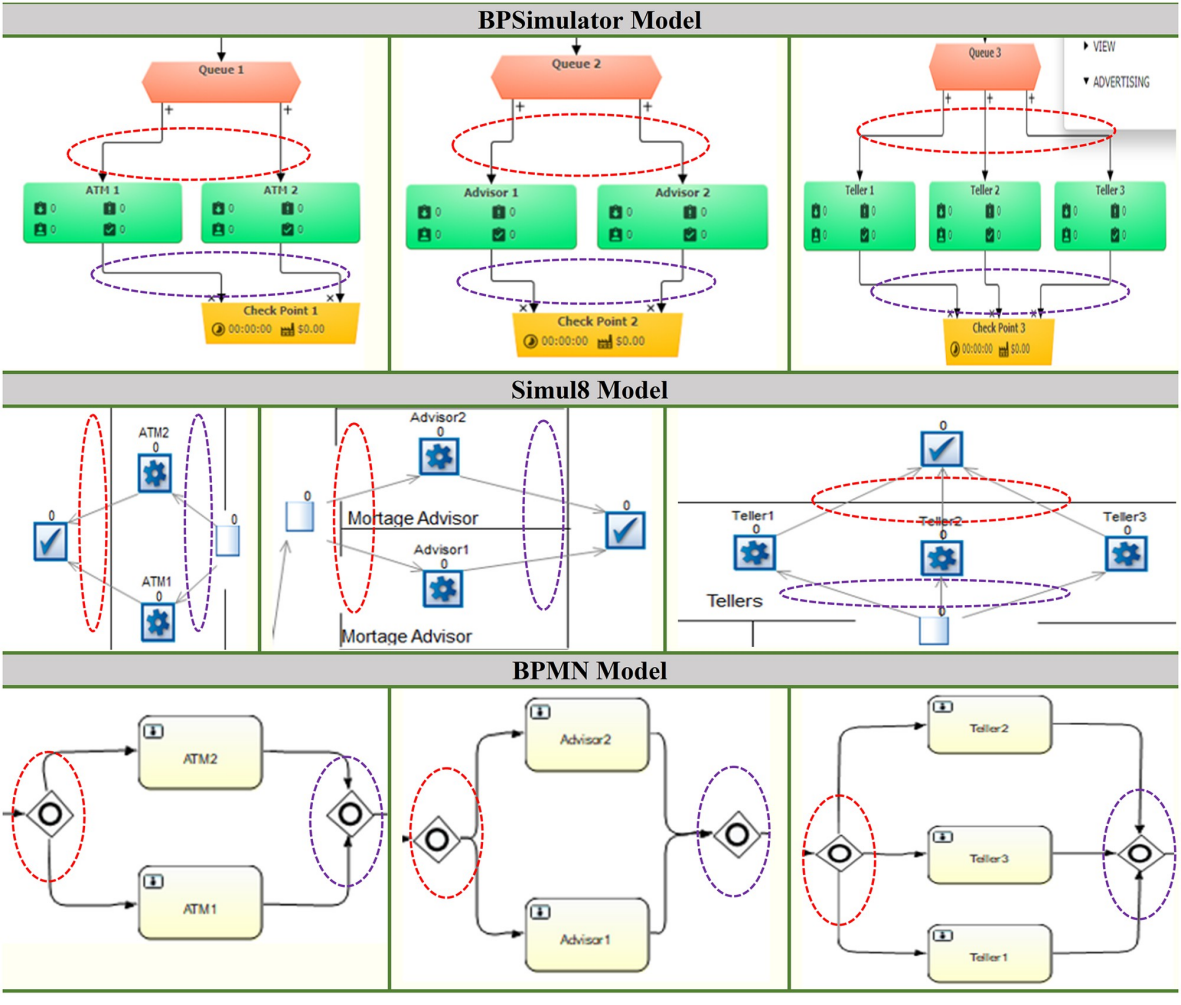
Three parts of bank model ATM, advisor and teller are shown. The connectors in Red oval in BPSimulator and Simul8 model is converted to opening parallel gateway. The connectors in purple oval is closing gateway.

The increase in number is due to the connector’s junction in the model, which is translated to the parallel gateway in BPMN model. Fig 12 shows first part of connectors that is converted to the parallel gateway. Fig 13 shows other three parts of the bank model where the connectors are separating the activities of ATM, teller and mortgage advisor. This is replaced by the parallel gateway notation highlighted in red dotted oval in BPMN diagram. The other dotted oval in purple shows the joining of the parallel gateway as for gateways in BPMN there are opening as well as closing gateway.

**Fig 14:**
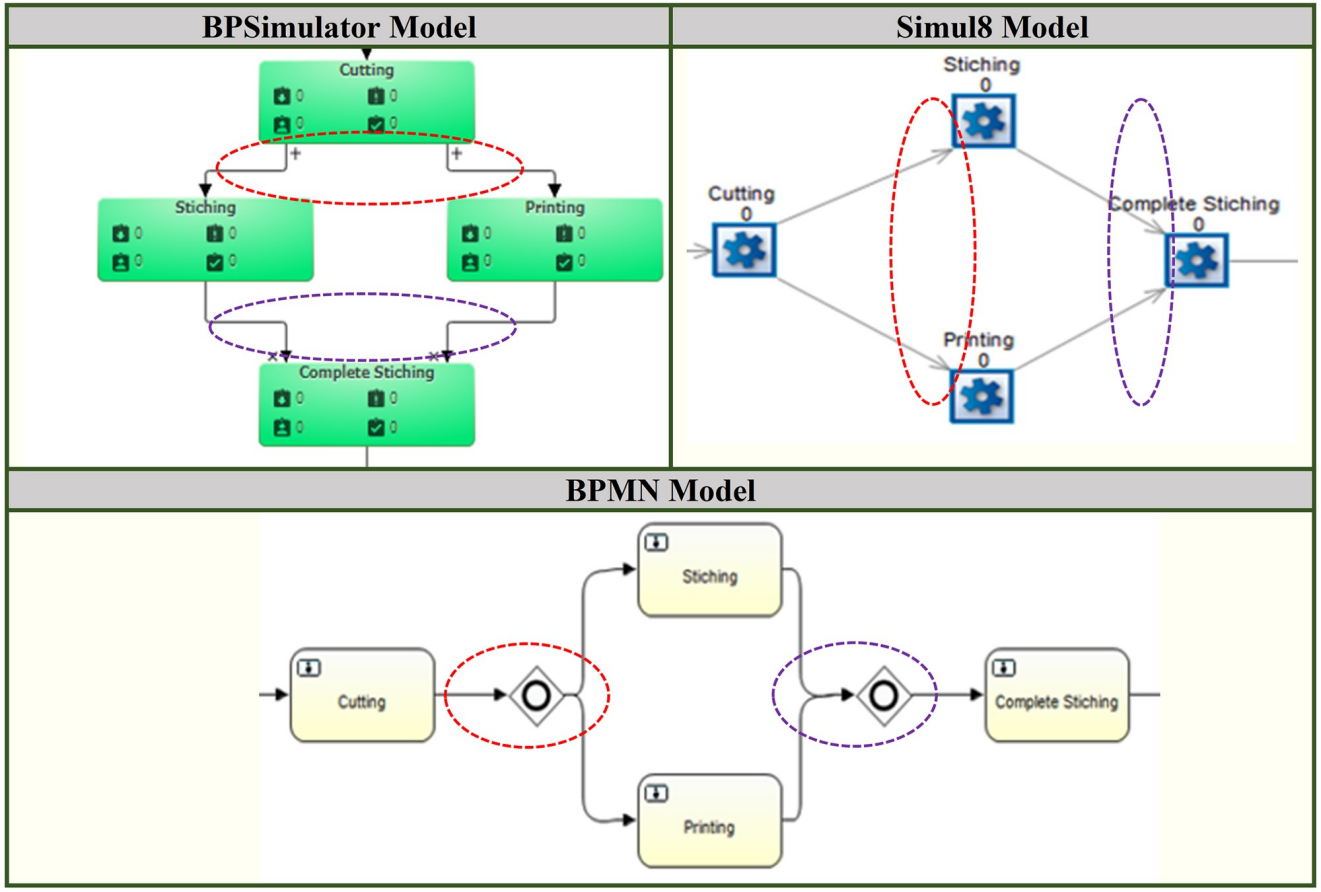
Part of shirt manufacturing model shows red and purple dotted oval in BPSimulator and Simul8 are converted to parallel gateways opening and closing in BPMN model.

A shirt-manufacturing model is built to test the framework with missing notations in models. In this model queue is not used in case of Simul8 model and checkpoint in case of BPSimulator is not used. The reason for this is to test the framework with the missing notations. This is also successfully converted to the final BPMN. The notations that are not used are not mapped or shown in the final output. Fig 14 shows the shirt manufacturing simulation model part from both BPSimulator and Simul8. The red dotted oval is connectors in both models and parallel gateway in BPMN model while purple dotted oval shows the connectors in both models but closing gateways in BPMN model.

## Conclusion and future work

Different approaches or graphical representation techniques are available to illustrate the business process. At first, various techniques were discussed, including flowcharts, Petri nets, data flow diagrams, role activity diagrams, business use cases, and BPMN. After discussing these approaches, it is clear that the BPMN is the most effective way to represent a business process because it is simple to use and understand. After studying the change through simulation, business analysts can make judgments with the help of BPR. Today, a variety of tools is available for transforming the BPMN into a simulation model. These tools are useless because of interoperability problems and a lack of advanced process simulation. The procedure of transforming the BPMN to a simulation model raises the concept for the opposite process after looking into the specifics of these tools. No tools available that offer a simulation model to BPMN reverse process. Reverse engineering was used to investigate the BPMN conversion to a simulation model in simulation tools like Simul8 and BPSimulator. The simulation model notation was then mapped to the BPMN notation, which is a language of notations used to represent business processes, using the rules of the re-engineering process. It was decided to create an architecture that would map the simulation and BPMN notations. The simulation model, which comprises an exhaustive definition of the business process, will be entered by the user. Then the system will convert the simulation model into a BPMN diagram by mapping the notations. After mapping the notations of the simulation model to the BPMN diagram, an XML file is generated, which contains all the notations of the BPMN diagram. When this file is opened in the eclipse shows the BPMN diagram of the business process.

This research project aims to shorten the duration of the dual effort that involves first constructing a BPMN model and then a simulation of the process during BPR. As was already said, the BPMN simulation tools can only mimic processes up to a certain level; they cannot simulate complex processes. By initially creating a simulation model, our converter will assist the analysts in validating and verifying the model using the supplied data. The decision will be based on the output analysis, and any changes that are necessary can be made in either a complicated or basic simulation model. Once the desired outcomes are obtained for the business process to be reengineered, the BPMN model will automatically be built with only one click, saving time and resources.

According to Standish Group Report published in 2015 more than 50% of the software projects are either challenged or failed [33]. The reason for failure of software projects include unsatisfaction from customer due to poor understanding of the requirements. Requirements gathering and understanding is the main factor for the success or failure of the projects [34]. Any business process can be understood in order to better grasp the requirements, and as a result, communication between clients and engineers improves. BPMN is one of the industry-standard methods for business analysis and modelling [35]. The requirements engineering community, however, has not done a good job of producing a manual for using standardized tools for engineering software requirements. Verification and validation of the requirements can help reduce the chances of failure or changes in the project. As the simulation model is a dynamic representation of the system that is verified using various methods like we can make decision on historic data input, the simulation model can tell us the required change is useful or not. After the simulation model has been created, it is validated using real data input to obtain a comprehensive understanding of the entire process. As a result, creating a BPMN using this model is more proven and confirmed than creating one by studying the process. Furthermore, there is XML generated with BPMN during the conversion process that contain entities and its attributes. This XML will be used to build data model of the business process that will lead to generate micro services and interfaces for the process. This will help the software developers by saving time and cost in building the micro services and interfaces.
